# Hydrothermal synthesis of In_2_O_3_ nanoparticles hybrid twins hexagonal disk ZnO heterostructures for enhanced photocatalytic activities and stability

**DOI:** 10.1186/s11671-017-2233-3

**Published:** 2017-07-25

**Authors:** Hairui Liu, Haifa Zhai, Chunjie Hu, Jien Yang, Zhiyong Liu

**Affiliations:** 1 College of Physics and Materials science, Henan Normal University, Xinxiang, 453007 People’s Republic of China; 2 Henan Key Laboratory of Photovoltaic Materials, Henan Normal University, Xinxiang, 453007 People’s Republic of China

**Keywords:** ZnO, In_2_O_3_, Heterostructures, Photocatalytic efficiency

## Abstract

In_2_O_3_ nanoparticles hybrid twins hexagonal disk (THD) ZnO with different ratios were fabricated by a hydrothermal method. The as-obtained ZnO/In_2_O_3_ composites are constituted by hexagonal disks ZnO with diameters of about 1 μm and In_2_O_3_ nanoparticles with sizes of about 20–50 nm. With the increase of In_2_O_3_ content in ZnO/In_2_O_3_ composites, the absorption band edges of samples shifted from UV to visible light region. Compared with pure ZnO, the ZnO/In_2_O_3_ composites show enhanced photocatalytic activities for degradation of methyl orange (MO) and 4-nitrophenol (4-NP) under solar light irradiation. Due to suitable alignment of their energy band-gap structure of the In_2_O_3_ and ZnO, the formation of type п heterostructure can enhance efficient separation of photo-generate electro-hole pairs and provides convenient carrier transfer paths.

**Electronic supplementary material:**

The online version of this article (doi:10.1186/s11671-017-2233-3) contains supplementary material, which is available to authorized users.

## Background

In recent years, environmental pollution and energy shortage have created serious social and economic issues for human society. Semiconductor-based photocatalysis has been widely employed as a highly efficient technique to overcome these issues [1–3]. Among these semiconductor metal oxides, zinc oxide (ZnO) has been recognized as a promising photocatalyst owing to its outstanding electrical and optical properties, low cost, high biological safety, versatile shapes and structures, environment benign and strong photocatalytic degradation ability of organic pollutants under UV light. However, ZnO with a wide band gap (Eg = 3.3 eV) can only be activated by ultraviolet (UV) light, which restricts its practical applications for solar energy [4–8]. Another main drawback of ZnO is rapid recombination of photo-induced electron-hole pairs, which results in the low quantum yield for any photocatalytic reactions [9–12]. Therefore, how to extend absorption edge of ZnO to visible light region for the utilization of about 43% solar spectrum meanwhile suppress the photo-generated electron-hole pairs recombination is still a great challenge for scientists. Various modification strategies to activate ZnO photocatalysis under visible light have been employed in the past few years, including sensitization, semiconductor coupling and doping. An efficient strategy is coupling ZnO with another narrowband-gap semiconductor (e.g. CdS [13], CdSe [14], Cu_2_O [15], C_3_N_4_ [16], ZnFe_2_O_4_ [17], Ag_3_PO_4_ [18], CuInS_2_ [19], AgBr [20] and BiVO_4_ [21]) to form ZnO/narrow-band-conductor type п heterostructures. The formation of type II heterostructures has been recognized as an attractive route to overcome the limitations of ZnO because it promotes efficient charge separation, enlarges the effective contact interfaces and improves the optical absorption [22, 23].

In_2_O_3_ with a band gap of 2.56 eV has been proved as efficient sensitizer to extent the light absorption spectra by coupling other semiconductor. Also, its valence and conduction band alignments are staggered relative to those of ZnO [24, 25]. A lot of researches on In_2_O_3_-ZnO composite have been reported for degradation of organic compounds and hydrogen production by photocatalysis [26–28]. These results show that the incorporation of In_2_O_3_ in ZnO nanostructure can remarkably inhibit recombination of photo-generated electron-hole pairs and thus improve the photocatalytic activity. To the best of our knowledge, there has rarely been reported on the fabrication and improvement ZnO photocatalytic activities and stability by In_2_O_3_ nanoparticles hybrid.

In this paper, In_2_O_3_ nanoparticles hybrid THD ZnO with different ratios were fabricated by a hydrothermal method. The microstructure and optical properties of ZnO/In_2_O_3_ heterostructures were examined. The photocatalytic activity and photo-stability of ZnO/In_2_O_3_ composites were evaluated by MO and 4-NP under light irradiation. Finally, the charge transfer and probable photocatalytic mechanism have been discussed and proposed on the basis of optical characterization, band gap structure and reactive species reaction.

## Experimental

### Formation of ZnO/In_2_O_3_ heterostructure

First, 0.1 mol of ZnAc and a specific molar of In(NO_**3**_)_**2**_ with a designed atom percent of In to Zn (about 2.0, 5.0, 8.0, 12.0 and 15.0 atom%) were dissolved in 50 ml deionized water to form a clear solution. Then, 15 ml of triethanolamine (TEA) was dropwise into the above solution under magnetically stirring. After that, the mixed solution was heated at 90 °C for 4 h, the obtained precipitates were centrifuged and washed by deionized water and ethanol for several times and dried in an oven at 60 °C. The final ZnO/In_2_O_3_ composites were thus obtained by annealing at 200 °C for 1 h. According to the In/Zn molar ratios of 0, 2, 5, 8, 12 and 15%, the composites were marked as Zn-In-0, Zn-In-1, Zn-In-2, Zn-In-3, Zn-In-4 and Zn-In-5, respectively. For comparison, pure In_2_O_3_ were also fabricated under the same condition.

### Characterization

The crystal structures were studied by powder X-ray diffraction (XRD) with a 0.154178 nm Cu-Kα radiation. The morphologies and size of the ZnO/In_2_O_3_ composites were measured by field emission scanning electron microscopy (FESEM; JSM-6700F, Japan). Chemical compositions were analyzed by X-ray energy-dispersive spectroscopy (EDS) equipped to the SEM. The detailed microstructures of samples were characterized by high resolution transmission electron microscopy (FE-SEM SUPRA™ 40). Chemical states of the samples were analyzed using X-ray photoelectron spectroscopy (XPS; PHI-5300, ESCA, USA). The UV-vis diffused reflectance spectra (UV-vis DRS) of samples were measured on a UV-3600 spectrophotometer. Photoluminescence (PL; Renishaw1000, UK) spectra were measured at room temperature using a He-Cd laser as the excitation light source at 325 nm. The •OH-trapping PL spectra was collected in 5 * 10^−3^ M terephthalic acid solutions containing 0.01 M NaOH solution with different irradiation time; the excitation wavelength was 325 nm.

### Photocatalytic test

The photocatalytic activities of the as-prepared samples were evaluated by the photocatalytic degradation of MO and 4-NP. The wavelength distribution of Xenon lamp was similar to that of solar light; thus, a 500 W Xenon lamp was employed as the light source. For each photocatalytic activity measurement, typically, 10 mg of the photocatalyst was dispersed in 50 ml of MO (5 mg/l) or 4-NP (1 mg/l) aqueous solution and then stirred in the dark for 30 min to achieve an adsorption-desorption equilibrium. The photocatalytic reaction was carried out by Xenon lamp as the solar light source with continuous stirring. At the given intervals, 3 mL of the aliquots was sampled and analyzed by recording variations in the absorption band (464 and 317 nm) in the UV-vis spectra of MO or 4-NP, respectively. To probe the photo-stability of the Zn-In-4 catalyst, cycle degradation was carried out. In this case, Zn-In-4 was repeatedly used, which was separated and collected by centrifugation. After being washed with water and ethanol for several times and dried at 60 °C overnight, the Zn-In-4 catalyst was reused with a fresh MO aqueous solution (5 mg/l) for subsequent reactions under the identical conditions.

Trapping experiments were performed to probe the main active species in the photocatalytic process. The experimental apparatus and procedures were identical to that of the photocatalytic activity tests except that different types of scavengers (1 mM) were added into the MO solution. Herein, a fluorescence technique was employed to detect the formation of free hydroxyl radicals (•OH) and terephthalic acid (TPA) was used as the probe molecule. In detail, the as-synthesized Zn-In-4 (0.025 g) was dispersed into 50 mL mixed solution of 0.25 mmol TPA and 1 mmol NaOH under magnetically stirring. After Xenon lamp (500 W) irradiation for 90 min, the supernatant of reaction solution was collected and examined by a FP-6500 fluorescence spectrophotometer with an excitation wavelength of 315 nm.

## Results and discussion

### Morphology and phase structure analysis

**Fig. 1:**
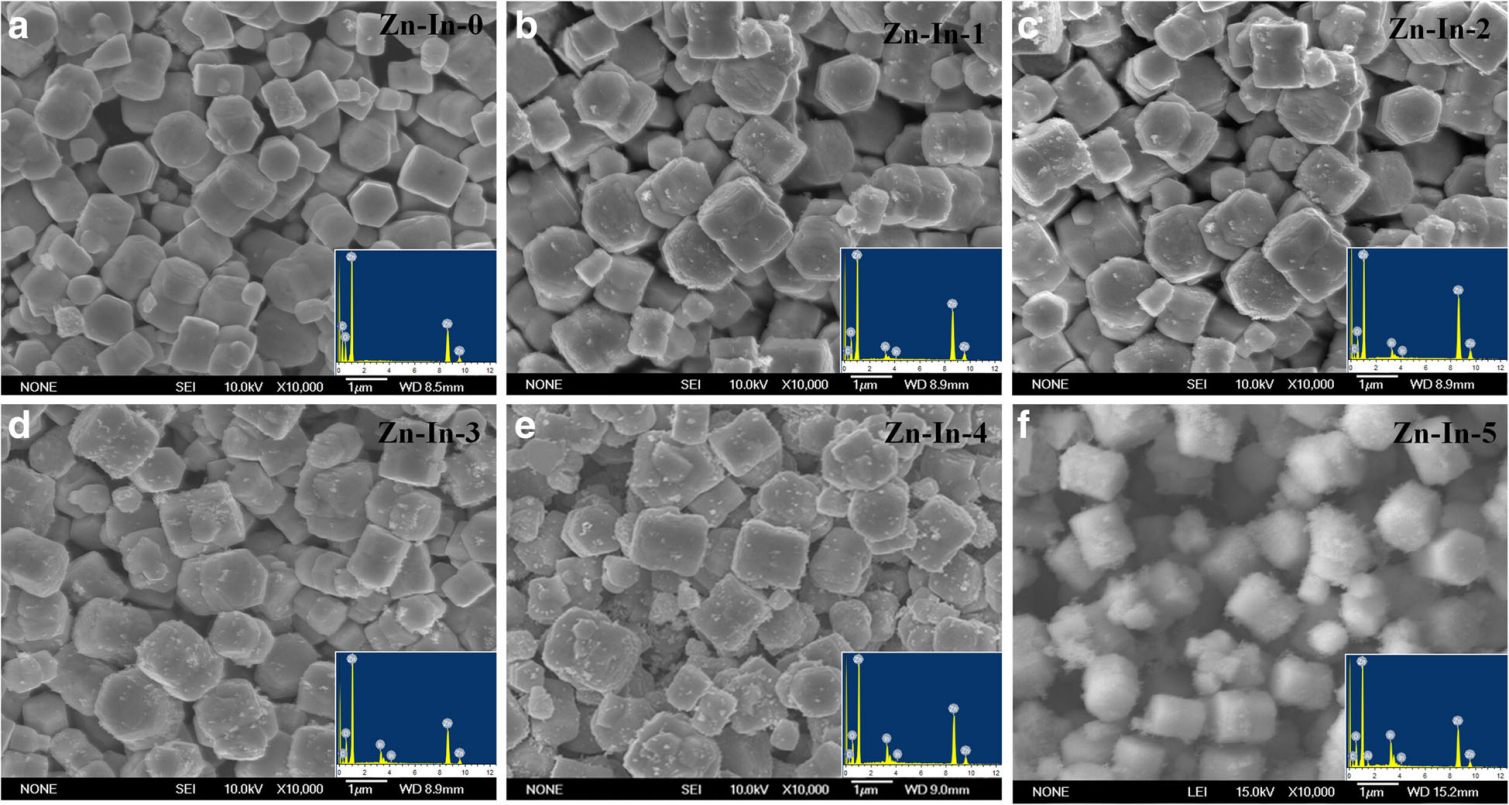
SEM images of ZnO/In_2_O_3_ composites with different In_2_O_3_ amount (**a**–**f**). The *insets* are the EDS spectra corresponding samples

Figure 1 gives the SEM images of fabricated ZnO/In_2_O_3_ composites with different loading amounts of In_2_O_3_. It can be clearly seen from Fig. 1a that the pure ZnO present twins hexagonal disk shape. The twins hexagonal disk have an average side length value of about 700–1000 nm, and the height of every disks is about 300–400 nm. It is clearly indicated in Fig. 1 that all samples retain the THD morphology and the size of the samples does not change with In_2_O_3_ content increasing. The only difference is that the amount of In_2_O_3_ nanoparticles on the surface of ZnO/In_2_O_3_ composites increase with the increasing of In(NO_3_)_3_ content. It should be mentioned that the In_2_O_3_ nanoparticles are uniformly distributed on the surface of each THD ZnO, There is rare aggregations even for higher In_2_O_3_ contents sample. The EDS spectra of ZnO/In_2_O_3_ samples (inserted to corresponding SEM image) were detected by dispersed samples onto a conductive carbon tape. Elemental zinc, oxygen, and indium are detected, and the corresponding weight and atomic percentages for the all samples are listed in Table 1.

**Table 1:** Weights and atomic percentages of elements in ZnO/In_2_O_3_ composites

Sample	Zn-In-0		Zn-In-1			Zn-In-2			Zn-In-3			Zn-In-4			Zn-In-5		
Element	Zn	O	Zn	O	In	Zn	O	In	Zn	O	In	Zn	O	In	Zn	O	In
Wt%	78.5	21.5	76.5	20.9	2.6	73.1	20.7	6.2	69.8	20.7	9.5	65.9	20.8	13.3	63.4	20.4	16.2
Atom%	47.3	52.7	47.3	52.8	0.9	45.4	52.4	2.2	43.7	52.9	3.4	41.6	53.6	4.8	40.6	53.5	5.9

**Fig. 2:**
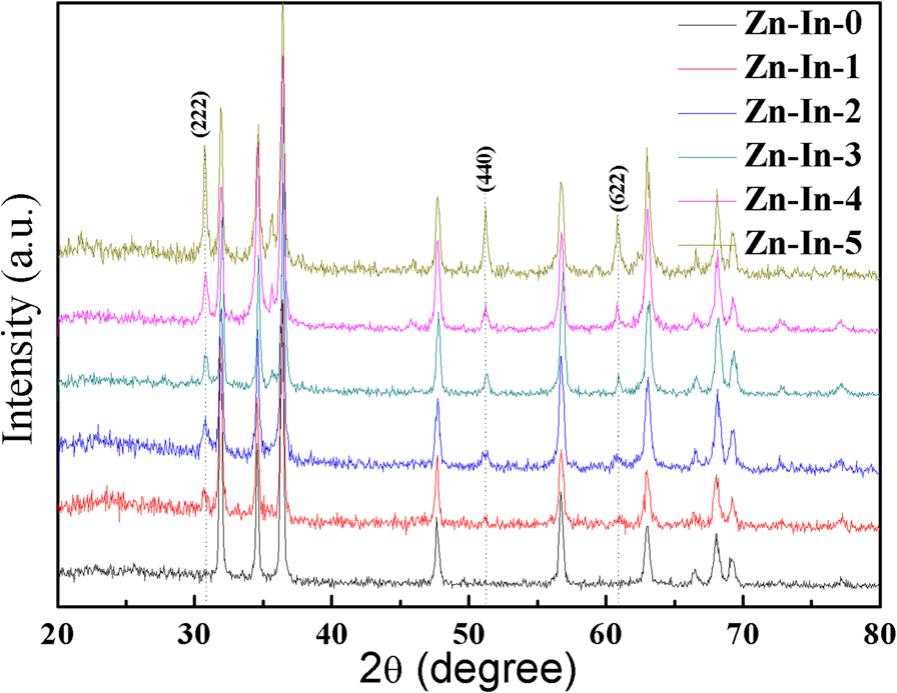
XRD patterns of ZnO/In_2_O_3_ composites with different In_2_O_3_ amount

Figure 2 presents the XRD patterns of the ZnO/In_2_O_3_ composites. For Zn-In-0 sample, all diffraction peaks match well with wurtzite ZnO structure (JCPDS 36–1451). For the ZnO/In_2_O_3_ heterostructure composites, three new characteristic peaks appear at 2θ values of 30.6, 51.1, and 60.7 can be indexed to (222), (440), and (622) crystal planes of body-centered cubic structure of In_2_O_3_ (JCPDS, No. 71–2194), respectively. However, with the molar ratio increasing of In:Zn in ZnO/In_2_O_3_ composites, the intensities of typical characteristic peaks indexed to In_2_O_3_ increase. No characteristic peaks for other impurities are observed, confirming that the successfully fabricated ZnO/In_2_O_3_ composites have high purity.

**Fig. 3:**
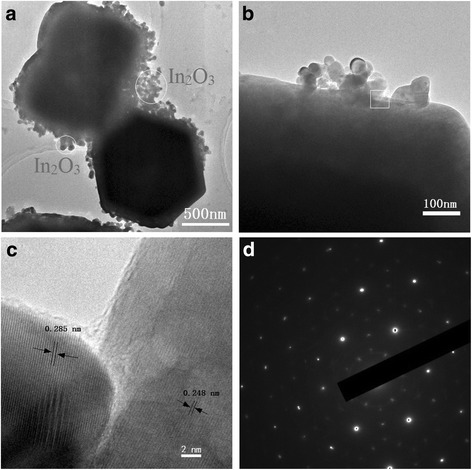
TEM image (**a**) and HR-TEM images (**b**, **c**) of Zn-In-4 sample

To further obtain the morphology and structure information, Fig. 3 presents HR-TEM images of Zn-In-4 sample. It can be found that hexagonal disk structures have a diameter of about 800 nm and the surfaces are covered with In_2_O_3_ nanoparticles_._ It is obvious that ZnO/In_2_O_3_ heterostructure is composed of twinned hexagonal disks ZnO and In_2_O_3_ nanoparticles. Fig. 3(b) indicates the edges of twinned hexagonal disks covered by nanoparticles with diameters of 20–50 nm. The HR-TEM image of the white-square area of Fig. 3b is shown in Fig. 3c, a clearly distinguished interface can be observed from Fig. 3c. The spacing with 0.248 nm is consistent with the inter-planar spacing of the (002) planes of hexagonal ZnO phase [12]. The left part clearly exhibits the In_2_O_3_ (222) facets with a spacing value of 0.285 nm, which is consistent with the reported value [24]. The good crystalline quality and the sharp interface between ZnO and In_2_O_3_ would be advantageous for the separation of the photo-generated charge carriers. Figure 3d is the selected area electron diffraction (SAED) pattern of the interface, which consists of two sets of zone diffraction spots. These mixed diffraction patterns further indicate the presence of the In_2_O_3_ crystalline nucleus on the interface of ZnO hexagonal disk.

### XPS analysis

**Fig. 4:**
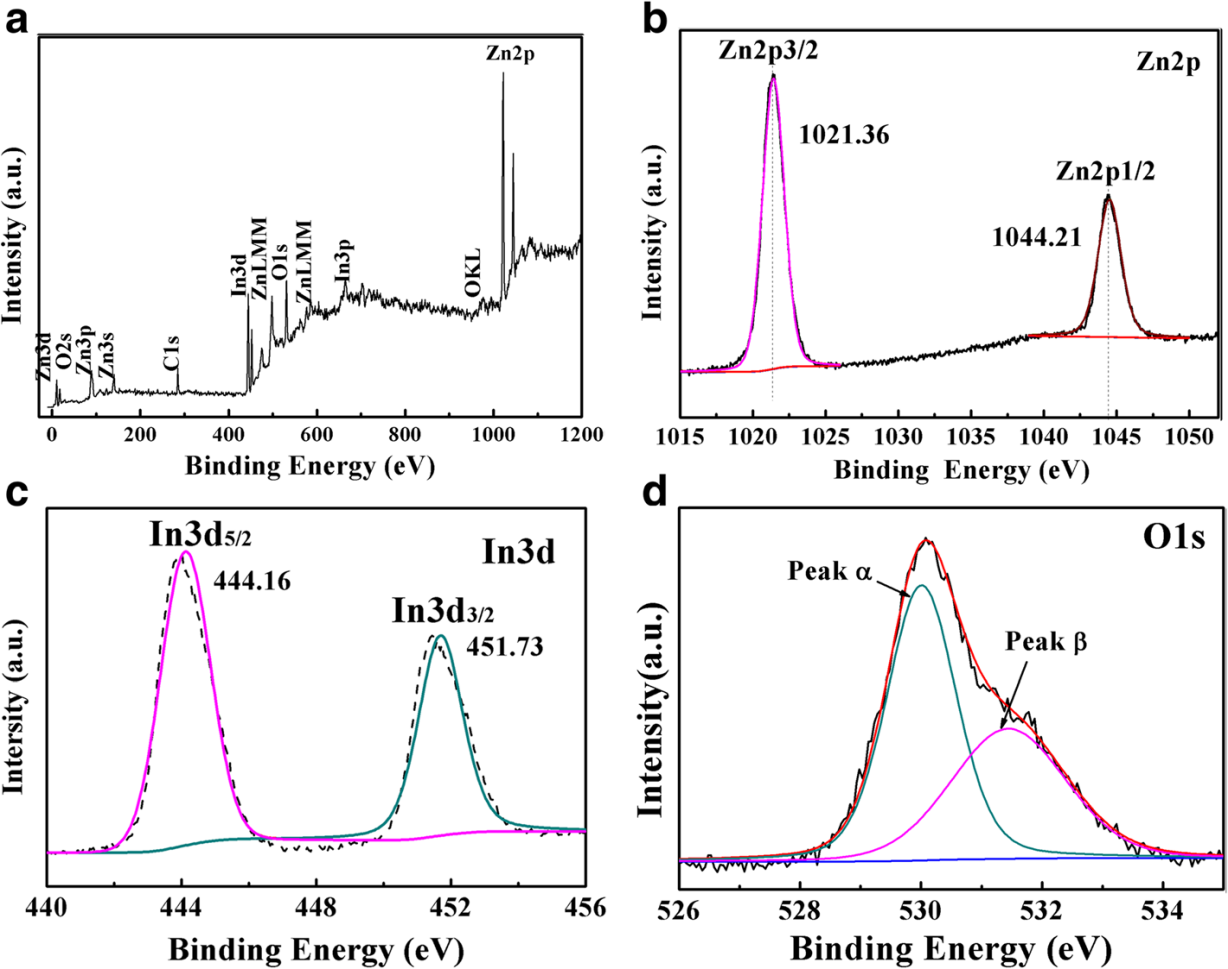
XPS survey spectrum of Zn-In-4 and corresponding high-resolution XPS spectra: (**b**) Zn2p, (**c**) In3d and (**d**) O1s. The units of Fig. 4 (**a**),(**b**),(**d**) should be "Binding Energy" rather than "Banding Energy". The replaceable Fig. 4 (**a**), (**b**), (**d**) shown in attachment

XPS measurement was carried out to further identify the surface elemental and chemical states of ZnO/In_2_O_3_ composites. The survey spectra (Fig. 4a) reveal the presence of the Zn2p, In3d, O1s and C1s energy regions. The high-resolution Zn2p spectrum in Fig. 4b showed two major fitting peaks centered at 1044.21 and 1021.36 eV, which are assigned to Zn2p1/2 and Zn2p3/2, respectively, indicating the Zn (II) oxidation state in ZnO [20]. In terms of the In 3d spectrum (Fig. 4c), there are two characteristic peaks centered at 444.16 and 451.73 eV that can be attributed to In 3d_5/2_ and In 3d_3/2_, which indicate the presence of In^3+^ in the ZnO/In_2_O_3_ composites [27, 29]. In the O 1 s XPS spectrum (Fig. 4d, the asymmetric profile can be divided to two symmetrical peaks centered at 530.06 and 531.74 eV, respectively. The peak located at 530.06 eV is assigned to lattice oxygen binding with In and Zn (denoted as In-O and Zn-O). In addition, the peak centered at 531.74 eV is associated with the surface-absorbed oxygen species [26, 30]. Many documents have recorded that the surface oxygen species can produce primary active superoxide radicals and hydroxyl radicals, which are capable to trap photo induced electrons and holes to enhanced photocatalytic activities [8, 31].

### Optical characteristics

**Fig. 5:**
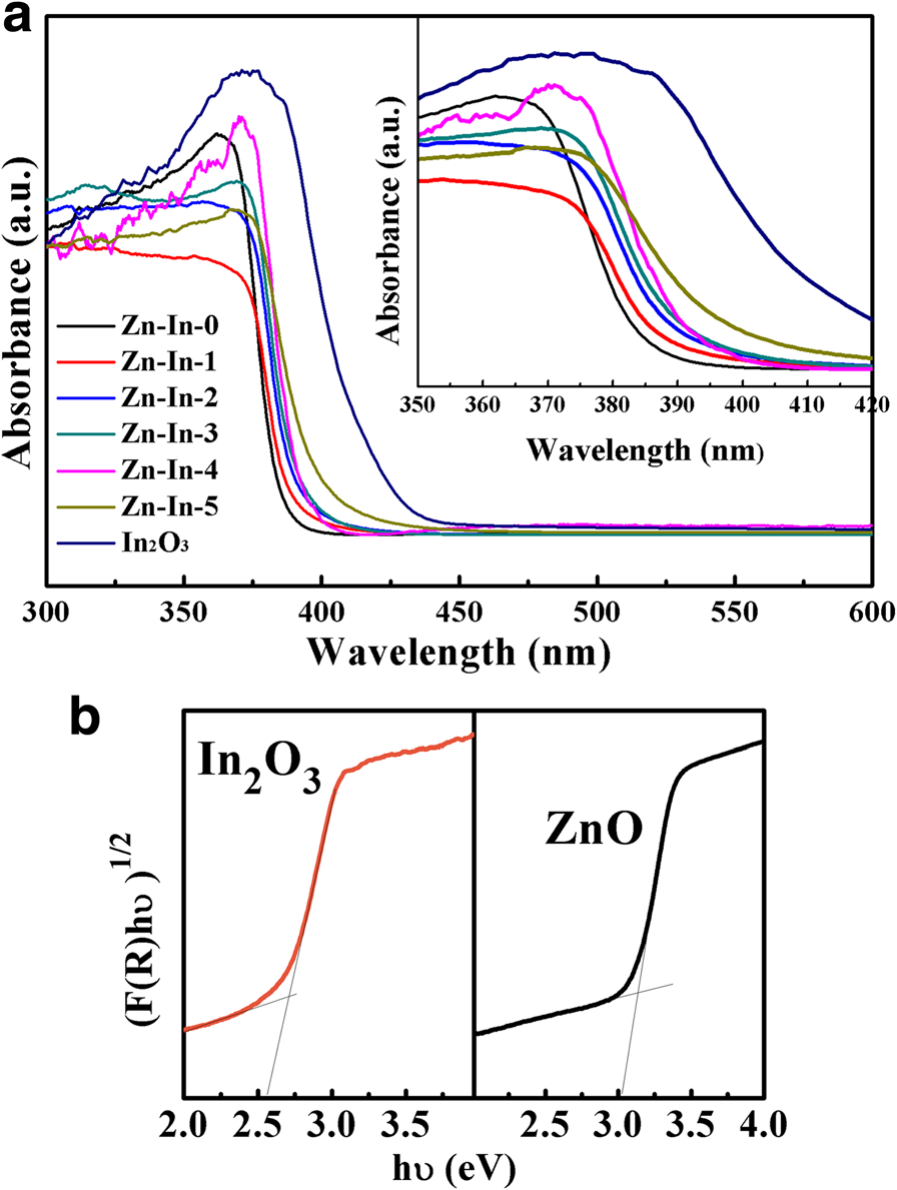
**a** UV-vis diffuse reflectance spectra of the prepared composites and **b** band gap energy of pure ZnO and In_2_O_3_ samples

Figure 5a shows the UV-vis diffuse reflectance spectra (UV-vis DRS) of the obtained ZnO/In_2_O_3_ composites. The bare ZnO show a broad absorbance with an absorption edge at 385 nm owing to the intrinsic wide band gap, whereas the cut off wavelength of pure In_2_O_3_ nanoparticles located at 450 nm. With the increase of In_2_O_3_ content from 2 to 15 at%, the absorption band edges of samples shifts from 380 to 420 nm and the color of the prepared samples also from whitish-yellow to brilliant yellow. This result implies that In_2_O_3_ nanoparticles successfully incorporate into ZnO. The inset is the magnified view of UV-vis DRS with the wavelength from 350 to 420 nm. According to Kubelka-Munk method [32], the band gap energy values for ZnO and In_2_O_3_ is estimated 3.18 and 2.75 eV, respectively [25, 33]. The plots of (F(R)hν)^1/2^ vs. hν of the photocatalysts are presented in Fig. 5b.

**Fig. 6:**
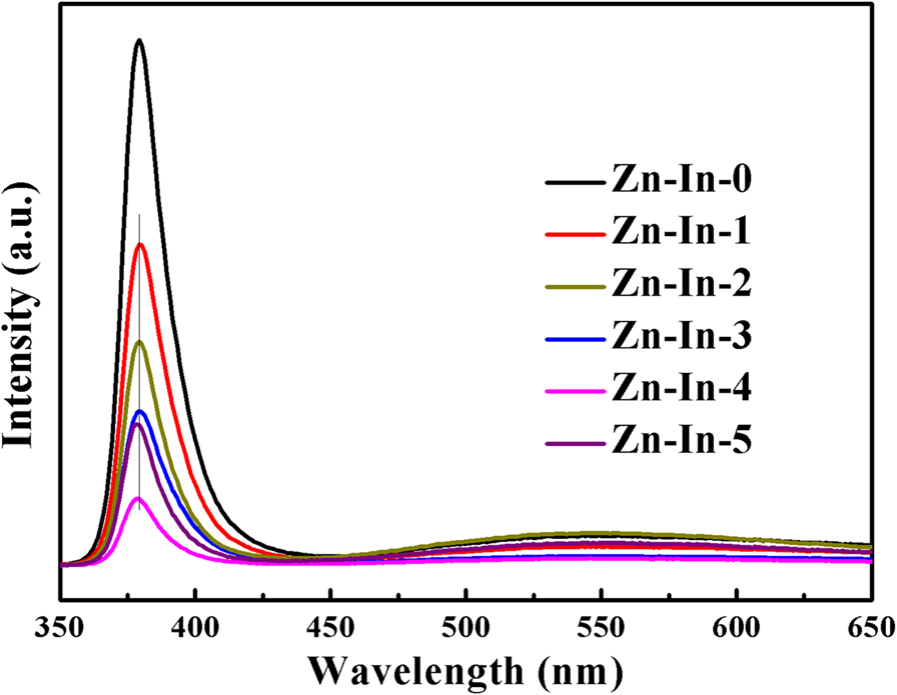
PL spectra of ZnO/In_2_O_3_ composites with different In_2_O_3_ amount

Photoluminescence (PL) technique is widely used to investigate the migration, transfer and separation efficiency of the photo-induced electrons-holes pairs in a photocatalyst. The higher PL intensity indicates the faster recombination rate of the photo-generated charge carriers; the fewer photogenerated electrons and holes participated in the photocatalytic redox reactions results in lower photocatalytic activity [15, 34, 35]. Consequently, in order to investigate the effect of In_2_O_3_ nanoparticles to ZnO, the PL emission spectra of ZnO/In_2_O_3_ composites with different contents of In_2_O_3_ were measured at room temperature under the excitation wavelength of 325 nm, as shown in Fig. 6. In this investigation, Zn-In-0 exhibits a strong UV luminescence emission peak centered at about 380.0 nm. The UV emission is attributed to the near band edge emission of ZnO. After the modification of In_2_O_3_, the emission intensity of ZnO/In_2_O_3_ samples dropped significantly. This result indicates that the recombination efficiency of photo-induced electron-hole pairs can be effectively inhibited through the formation of heterojunction structure. However, the Zn-In-4 samples showed the lowest intensity of PL emission peak, which means the Zn-In-4 has highest photocatalytic activity for all ZnO/In_2_O_3_ samples.

### Photocatalytic activity

**Fig. 7:**
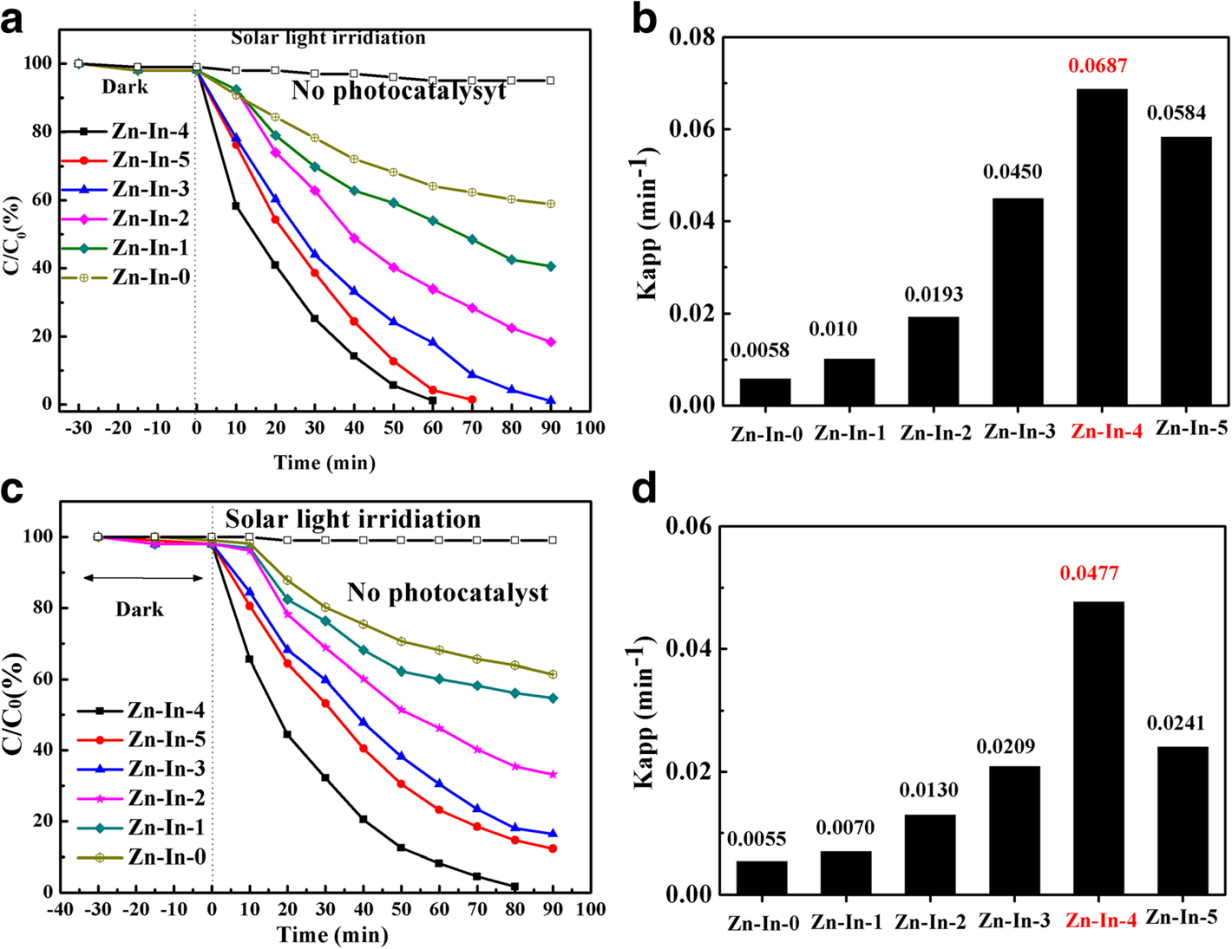
Photocatalytic degradation curves and Kinetic linear simulation rate constants of MO (**a**, **b**) and 4-NP (**c**, **d**) by Zn-In-0, Zn-In-1, Zn-In-2, Zn-In-3, Zn-In-4, Zn-In-5

The photocatalytic activities of ZnO/In_2_O_3_ samples were evaluated by degradation of MO and 4-NP under simulated solar irradiation. As illustrated in Fig. 7, self-degradation of MO and 4-NP is negligible without addition of photocatalysts, indicating these two types of organic dyes are photochemical stable. Figure 7a shows the degradation curves of MO on the ZnO/In_2_O_3_ samples. Under solar irradiation, only 35% of MO is decomposed after 90 min for Zn-In-0 photocatalysts, which attributed to its higher band gap energy. In comparison, the degradation rate of MO is about 64 and 82% for the Zn-In-1 and Zn-In-2 after 90 min of treatment, respectively. MO can be almost degraded thoroughly after 90 min treatment by Zn-In-3, 70 min by Zn-In-5, and only 60 min by Zn-In-4 composite photocatalyst. The temporal evolution of spectral changes accompanying the photodecomposition of MO over as-prepared Zn-In-4 is shown in Additional file 1: Figure S1. The characteristic absorption peak intensity of MO at 664 nm gradually decreased with the increase of irradiation time and the color of MO-containing solution was also changed from initial lemon yellow to almost transparent color after 60 min reaction, indicating that the MO have been completely decomposed during the photocatalytic process. According to the apparent pseudo-first-order kinetics equation, relative rate constants k_app_ for different catalysts are calculated and summarized in Fig. 7b. Their corresponding rate constants (*k*) are determined as 0.0058, 0.010, 0.0193, 0.0450, 0.0687 and 0.0584 min^−1^ for Zn-In-0, Zn-In-1, Zn-In-2, Zn-In-3, Zn-In-4, Zn-In-5, respectively. It can be found that rate constants k is first increase and then decrease with the increasing of In_2_O_3_ content in of ZnO/In_2_O_3_ composites. Zn-In-4 shows the highest photocatalytic activity. 4-NP was selected as another typical target compound for evaluating the photocatalytic activity of ZnO/In_2_O_3_ composites, and the photocatalytic degradation curves of 4-NP by ZnO/In_2_O_3_ composites are shown in Fig. 7c, d. With the In_2_O_3_ content increasing, the 4-NP degradation rate firstly increases and then decreases. Furthermore, the highest degradation rate is obtained from Zn-In-4 sample with almost 100% of 4-NP removal after 80 min solar light irradiation. The rate constants *k* of Zn-In-4 is about 12 times higher than Zn-In-0. Based on the above analysis, we can conclude that the photocatalytic activity of ZnO is enhanced significantly by In_2_O_3_ nanoparticles hybrid. With molar ratio increasing of In_2_O_3_ to ZnO, the degradation efficiency is firstly increased and then decrease, implying that the optimal loading amount of In_2_O_3_ is important for enhancing photocatalytic activity of ZnO/In_2_O_3_ composites.

As we all known, the mass ratio of components has a great effect on the photocatalytic performance in heterostructural photocatalyst system [27, 36]. With mass ratio increasing of In_2_O_3_ in ZnO/In_2_O_3_ composites, there was no significant difference in the degradation tendency of MO and 4-NP, and appears the maximum degradation efficiency for the sample Zn-In-4. However, PL intensities of the samples show an opposite variation tendency. This result indicates that an appropriate amount of In_2_O_3_ in the composites was beneficial to the fast separation of photo-generated charge carriers and thus enhanced the photocatalytic activity [5, 37].

**Fig. 8:**
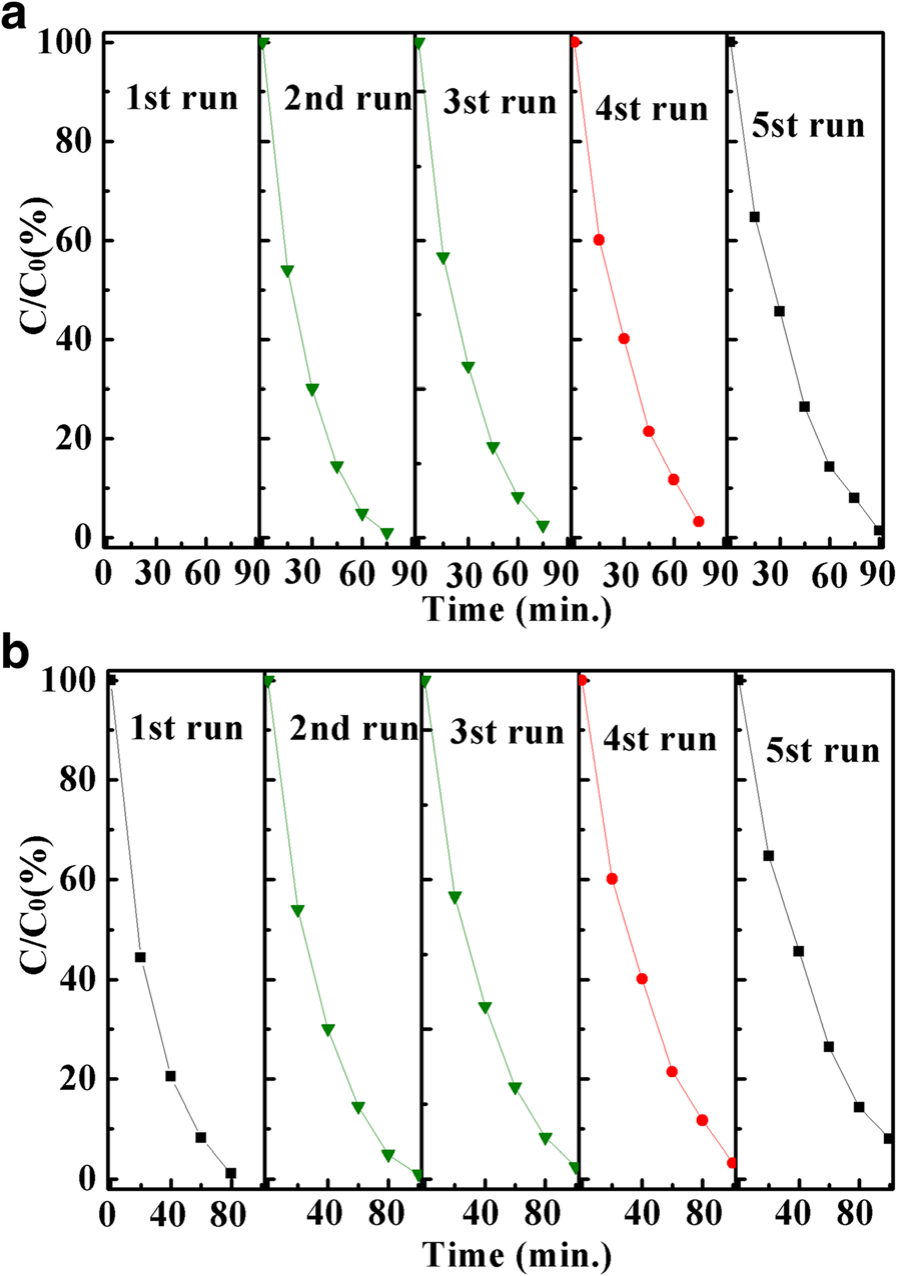
Recycling experiments of solar light photocatalytic degradation of MO (**a**) and 4-NP (**b**) over the Zn-In-4

It is well known that ZnO has poor stability in degradation of organic pollutants. So, to investigate the photo-stability and repeatability of ZnO-base photocatalyst is very important for photocatalytic performance. The recycling experiments were carried out by the degradation of MO (Fig. 8a) and 4-NP (Fig. 8b) solutions over Zn-In-4 under solar light irradiation. The degradation efficiency of MO drops from 99.7 to 82.6% and 4-NP from 99.5 to 85.4% after five cycles. There has a small variation in the degeneration efficiently of Zn-In-4 for different dyes after five recycling tests under solar light. In addition, the crystal structure as well as morphology of Zn-In-4 has no discernible change before and after five recycling tests under solar light irradiation. So, Zn-In-4 c is stable in photodegradation of organic pollutants.

### Proposed photocatalytic mechanism

**Fig. 9:**
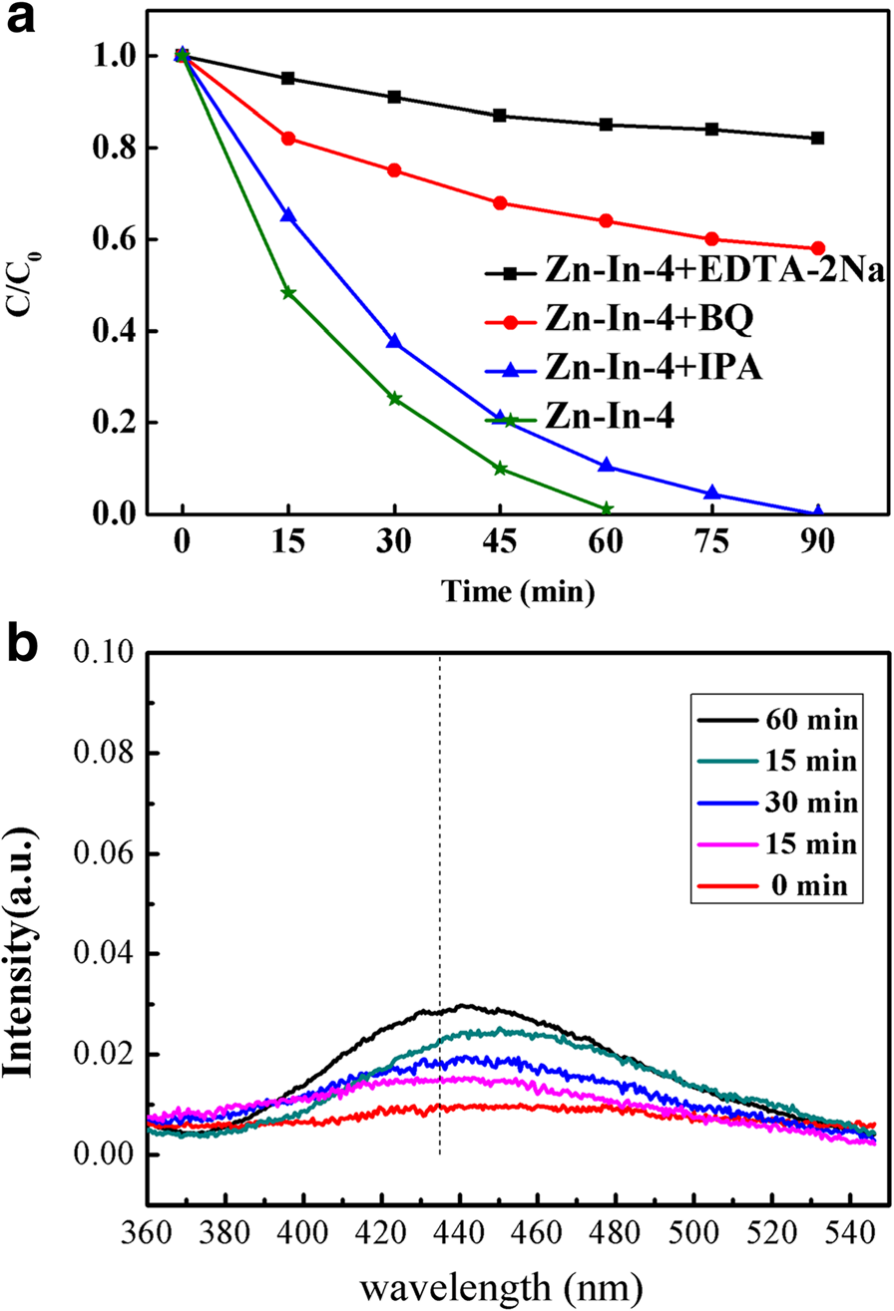
Trapping experiment of active species during the photocatalytic degradation of MO over Zn-In-4 under solar light irradiation (**a**) and the OH-trapping PL spectra over Zn-In-4 sample (**b**)

As been well known, it is important to investigate the active species in the photocatalytic process in order to better understand the mechanism of photocatalysis. The photocatalytic degradation of dyes mainly involved several active radical species, such as hole (h^+^), superoxide anion radical (•O_2_^−^) and hydroxyl radicals (•OH) [19, 38]. To evaluate the roles of these active species, a series of quenchers were employed during photodegradation processes. Benzoquinone (BQ), disodium salt ethylenediaminetetraacetic acid (EDTA-2Na), isopropanol (IPA) were used as scavengers for •O_2_^−^, photogenerated holes, and •OH in degradation of MO, respectively. As shown in Fig. 9, under solar light irradiation, the photocatalytic activity of Zn-In-4 composite is greatly suppressed by the addition of BQ or EDTA-2Na, suggesting that both photo-generated •O_2_^−^ and holes are the main oxidative species and played a crucial role in the degradation process of MO. However, there are little changes of the photodegradation performance when IPA added into photocatalytic system, suggesting that the •OH has a very small effect on the photocatalytic reaction system. In order to probe the photoactive hydroxyl radicals (•OH), the OH-trapping photoluminescence (PL) spectra (as shown in Fig. 9b) over Zn-In-4 suspension were collected in which terephthalic acid was used as trapping reagents for •OH. It was clear that the emission peak at 426 nm appeared under illumination and the intensity of the emission peak present a little change with the illumination time [39, 40]. Consequently, it can be furtherly confirmed that the photocatalytic degradation of MO over the as-prepared ZnO/In_2_O_3_ composite was mainly governed by •O_2_^−^ and h^+^ rather than •OH under solar light irradiation.

Therefore, to fully understand the photocatalytic reaction mechanism occurring during the photodegradation of as-prepared ZnO/In_2_O_3_ composites, the band edge positions of the valence band (VB) and conduction band (CB) of both In_2_O_3_ and ZnO are necessary to be determined. For a semiconductor, the VB and CB can be calculated according to the empirical equation [41]:

1 $$ {E}_{CB}=X-{E}_C-\raisebox{1ex}{$1$}\!\left/ \!\raisebox{-1ex}{$2$}\right.{E}_{\mathrm{g}} $$

2 $$ {E}_{VB}={E}_{CB}+{E}_g $$

**Fig. 10:**
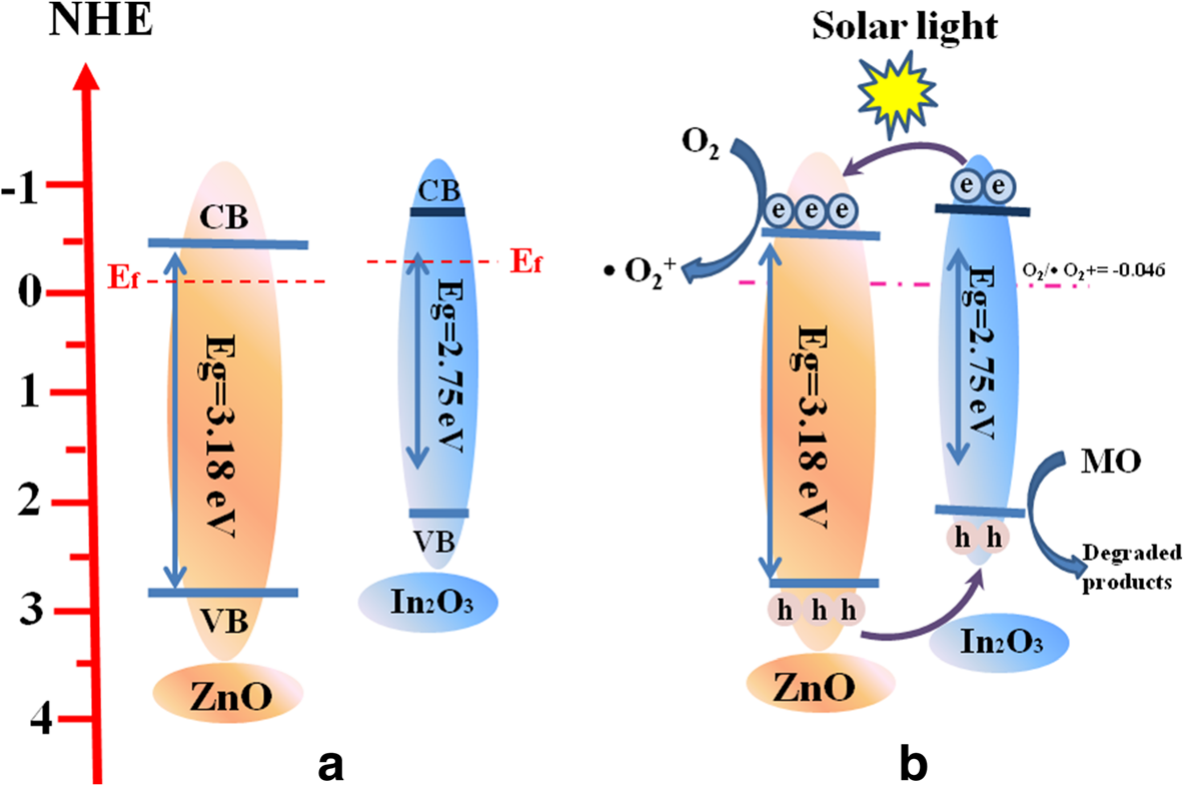
Schematic diagram of the energy alignment (**a**) and charge transfer in the ZnO/In_2_O_3_ composites under solar light irradiation (**b**)

Where E_VB_ is the valence band, *E*_*CB*_ is conduction band, *E*_*C*_ is the energy of free electrons with respect to normal hydrogen electrode (about 4.5 eV vs. NHE) and *Eg* is the band gap of the semiconductor. X is the absolute electronegativity of the semiconductor, according to previous literatures, the values of *X* for In_2_O_3_ and ZnO were 5.24 and 5.94 eV [27, 42], respectively. Based on the result in Fig. 5(b), the band gap energies of In_2_O_3_ and ZnO are estimated as 2.75 and 3.18 eV, respectively. Given the equations above, the *E*_*CB*_ of In_2_O_3_ and ZnO are estimated to be −0.635 and −0.15 eV, respectively; while the *E*_*VB*_ of In_2_O_3_ and ZnO are estimated to be 2.12 and 3.03 eV, respectively. Figure 10a shows the energy band structure of ZnO/In_2_O_3_ heterostructure. The Femi energy level (E_f_) of In_2_O_3_ is more negative than that of ZnO [33, 43]. So, in order to achieve Fermi energy level equilibration in In_2_O_3_/ZnO heterojunction-type photocatalyst, the Fermi level of ZnO is maintained its position due to the pinning effect of wide-band semiconductor while the Fermi level of In_2_O_3_ could shift up until reaching equilibrium. Under solar irradiation, both In_2_O_3_ and ZnO absorb light, the electrons will be excited and migrates to the CBs and holes remain on the VB of both In_2_O_3_ and ZnO. The electrons on the CB of In_2_O_3_ could easily transfer to the CB of ZnO. Simultaneously, the holes in the VB of ZnO migrate into the VB of In_2_O_3_. The electrons left at the CB of ZnO reduce O_2_ to yield •O_2_^−^, which is a powerful oxidant for organic dyes degradation [15, 44]. Holes stored in the VB of In_2_O_3_ could directly oxidize the pollutants to harmless products, as shown in Fig. 10b. Based on the above analysis, we can conclude that photo-generated h^+^ and •O_2_^−^ are the primary active species to determine photocatalytic performance, while the enhanced photocatalytic activity is attributed to the efficient separation and transfer of the photo-generated carriers at the heterojuction interfaces driven by the well-matched band-structures of ZnO and In_2_O_3_.

## Conclusions

In summary, In_2_O_3_ nanoparticles hybrid THD ZnO with different ratios were fabricated via the hydrothermal process. Significantly, compared with pure ZnO, the fabricated ZnO/In_2_O_3_ exhibits much better photocatalytic activities for the degradation of MO and 4-NP under simulated solar light irradiation, which can be ascribed to the synergetic effect between ZnO and In_2_O_3_, including the maximum heterostructure interface with intimate contact and excellent solar light response in the composite, which both can enhanced photogenerated charge separation efficiency. This work could give insights into the importance of rational design of heterostructure systems and provide a potential method for the construction of efficient heterostructure photocatalysts with controllable sizes and space distributions.

## Additional file

Additional file 1: Figure S1 The temporal evolution of spectral of MO photodecomposition by Zn-In-4. (DOC 571 kb)

## References

[1] Cao SW, Low JX, Yu JG, Jaroniec M (2015). Polymeric photocatalysts based on graphitic carbon nitride. Adv Mater.

[2] Wenderich K, Mul G (2016). Methos, mechanism, and applications of photodeposition in photocatalysis: a review. Chem Rev.

[3] Li K, Peng BS, Peng TY (2016). Recent advances in heterogeneous photocatalytic CO2 conversion to solar fuels. ACS Catal.

[4] Wang CL, Tan X, Yan JT, Chai B, Li JF, Chen SZ (2017). Electrospinning direct synthesis of magnetic ZnFe_2_O_4_/ZnO multi-porous nanotubes with enhanced photocatalytic activity. Appl Surf Sci.

[5] Hong DY, Zang WL, Guo MX, Fu YM, He HX, Sun J, Xing LL, Liu BD, Xue XY (2016). High piezo-photocatalytic efficiency of CuS/ZnO nanowires using both solar and mechanical energy for degrading organic dye. ACS Appl Mater Interfaces.

[6] Liang YC, Lee CM (2016). Cosputtering crystal growth of zinc oxide-based composite films: from the effects of doping to phase on photoactivity and gas sensing properties. J App Phys.

[7] Zha R, Nadimicherla R, Guo X (2015). Ultraviolet photocatalytic degradation of methyl orange by nanostructured TiO2/ZnO heterojunctions. J Mater Chem A.

[8] Miao Y, Zhang HJ, Yuan S, Jiao Z, Zhu XD (2016). Preparation of flower-like ZnO architectures assembled with nanosheets for enhanced photocatalytic activity. J Colloid Interface Sci.

[9] Zhang Y, Xing ZP, Liu XF, Li ZZ, Wu XY, Jiang JJ, Li M, Zhu Q, Zhou W (2016). Ti3+ self-doped blue TiO2(B) single-crystalline nanorods for efficient solar-drive photocatalytic performance. ACS Appl Mater Interfaces.

[10] Zhang N, Xie SJ, Weng B, Xu YJ (2016). Vertically aligned ZnO-au@CdS core-shell nanorod arrays as an all-solid-state vectorial Z-scheme system for photocatalytic application. J Mater Chem A.

[11] Fang SM, Xin YJ, Ge L, Han CC, Qiu P, Wu LN (2015). Facile synthesis of CeO2 hollow structures with controllable morphology by template-engaged etching of Cu2O and their visible light photocatalytic performance. Appl Catal B-Environ.

[12] Mukhopadhyay S, Maiti D, Chatterjee S, Devi PS, Kumar GS (2016). Design and application of au decorated ZnO/TiO2 as a stable photocatalyst for wide spectral coverage. Phys Chem Chem Phys.

[13] Liang YC, Lung TW (2016). Growth of hydrothermally derived CdS-based nanostructures with various crystal features and Photoactivated properties. Nanoscale Res Lett.

[14] Wu Y, Xu F, Guo DF, Gao ZY, Wu DP, Jiang K (2013). Synthesis of ZnO/CdSe hierarchical heterostructure with improved visible photocatalytic efficiency. Appl Surf Sci.

[15] Zou XW, Fan HQ, Tian YM, Yan SJ (2014). Synthesis of Cu2O/ZnO hetero-nanorod arrays with enhanced visible light-driven photocatalytic activity. CrystEngComm.

[16] Chen DM, Wang KW, Xiang DG, Zong RL, Yao WQ, Zhu YF (2014). Significantly enhancement of photocatalytic performances via core-shell structure of ZnO@mpg-C3N4. Appl Catal B-Environ.

[17] Liang YC, Liu SL, Hsia HY (2015). Physical synthesis methodology and enhanced gas sensing and photoelectrochemical performance of 1D serrated zinc oxide–zinc ferrite nanocomposites. Nanoscale Res Lett.

[18] Dong C, Wu KL, Li MR, Liu L, Wei XW (2014). Synthesis of Ag3PO4-ZnO nanorod composites with high visible-light photocatalytic activity. Catal Commun.

[19] Yang YW, Que WX, Zhang XY, Xing YL, Yin XT, Du YP (2016). Facile synthesis of ZnO/CuInS2 nanorod arrays for photocatalytic pollutants degradation. J Hazard Mater.

[20] Song JM, Zhang J, Ni JJ, Niu HL, Mao CJ, Zhang SY, Shen YH (2014). One-pot synthesis of ZnO decorated with AgBr nanoparticles and its enhanced photocatalytic properties. CrystEngComm.

[21] Peng FP, Ni YR, Zhou Q, Kou JH, Lu CH, Xu ZZ (2017). Construction of ZnO nanosheet arrays within BiVO4 particles on a conductive magnetically driven cilia film with enhanced visible photocatalytic activity. J Alloy Compd.

[22] Liang YC, Lin TY, Lee CM (2015). Crystal growth and shell layer crystal feature-dependent sensing and photoactivity performance of zinc oxide–indium oxide core-shell nanorod heterostructures. CrystEngComm.

[23] Liang YC, Lung TW, Xu NC (2017). Photoexcited properties of tin Sulfide Nanosheet-decorated ZnO Nanorod Heterostructures. Nanoscale Res Lett.

[24] Lin ZJ, Zhu Q, Dong Y, Liu SH, Li JG, Li XD, Huo D, Zhang M, Xie M, Sun XD (2016). Synthesis and formation mechanisms of morphology-controllable indium-containing precursors and optical properties of the derived In2O3 particles. CrystEngComm.

[25] Espid E, Taghipour F (2017). Development of highly sensitive ZnO/In2O3 composite gas sensor activated by UV-LED. Sens Actuators B Chem.

[26] Wei HZ, Cui XZ, Wang X, Xie ML, Wang LQ, Zhang J (2017). Tian, hierarchical assembly of In_2_O_3_ nanoparticles on ZnO hollow nanotubes using carbon fibers as templates: enhanced photocatalytic and gas-sensing properties. J Colloid Interface Sci.

[27] Martha S, Reddy KH, Parida KM (2014). Fabrication of In2O3 modified ZnO for enhancing stability, optical behaviour, electronic properties and photocatalytic activity for hydrogen production under visible light. J Mater Chem A.

[28] Zhang F, Li XY, Zhao QD, Chen AC (2016). Facile and controllable modification of 3D In_2_O_3_ microflowers with In_2_S_3_ nanoflakes for efficient photocatalytic degradation of gaseous ortho-dichlorobenzene. J Phys Chem C.

[29] Xing YL, Que WX, Yin XT, He ZL, Liu XB, Yang YW, Shao JY, Kong LB (2016). In2O3/Bi2Sn2O7 heterostructured nanoparticles with enhanced photocatalytic activity. Appl Surf Sci.

[30] Mady AH, Baynosa ML, Tuma D, Shim JJ (2017). Facile microwave-assisted green synthesis of Ag-ZnFe2O4@rGO nanocomposites for efficient removal of organic dyes under UV- and visible-light irradiation. Appl Catal B-Environ.

[31] She P, Xu KL, He QR, Zeng S, Sun H, Liu ZN (2017). Controlled preparation and visible light photocatalytic activities of corn cob-like au-ZnO nanorods. J Mater Sci.

[32] Thangavel S, Thangavel S, Raghavan N, Krishnamoorthy K, Venugopal G (2016). Visible-light driven photocatalytic degradation of methylene-violet by rGO/Fe3O4/ZnO ternary nanohybrid structures. J Alloy Compd.

[33] Hong YZ, Jiang YH, Li CS, Fan WQ, Yan X, Yan M, Shi WD (2016). In-situ synthesis of direct solid-state Z-scheme V_2_O_5_/g-C_3_N_4_ heterojunctions with enhanced visible light efficiency in photocatalytic degradation of pollutants. Appl Catal B-Environ.

[34] Wang J, Xia Y, Dong Y, Chen RS, Xiang L, Komarneni S (2016). Defect-rich ZnO nanosheets of high surface area as an efficient visible-light photocatalyst. Appl Catal B-Environ.

[35] Christoforidisa KC, Montini T, Bontempi E, Zafeiratosc S, Jaénd JJD, Fornasieroa P (2016). Synthesis and photocatalytic application of visible-light active β-Fe_2_O_3_/g-C_3_N_4_ hybrid nanocomposites. Appl Catal B-Environ.

[36] Liu HR, Hu YC, He X, Jia HS, Liu XG, Xu BS (2015). In-situ anion exchange fabrication of porous ZnO/ZnSe heterostructural microspheres with enhanced visible light photocatalytic activity. J Alloy Compd.

[37] Jo WK, Natarajan TS (2015). Facile synthesis of novel redox-mediator-free direct Z-scheme CaIn_2_S_4_ marigold-flower-like/TiO_2_ photocatalysts with superior photocatalytic efficiency. ACS Appl Mater Interfaces.

[38] Tang H, Chang SF, Tang GG, Liang W (2017). AgBr and g-C3N4 CO-modified Ag_2_CO_3_ photocatalyst: a novel multi-heterostructured photocatalyst with enhanced photocatalytic activity. Appl Surf Sci.

[39] Zhou M, Yang H, Xian T, Li RS, Zhang HM, Wang XX (2015). Sonocatalytic degradation of RhB over LuFeO3 particles under ultrasonic irradiation. J Hazard Mater.

[40] Chen XX, Li R, Pan XY, Huang XT, Yi ZG (2017). Fabrication of In_2_O_3_-Ag-Ag_3_PO_4_ composites with Z-scheme configuration for photocatalytic ethylene degradation under visible light irradiation. Chem Eng J.

[41] Islam MJ, Reddy DA, Han NS, Choi J, Song JK, Kim TK (2016). An oxygen-vacancy rich 3D novel hierarchical MoS_2_/BiOI/AgI ternary nanocomposite: enhanced photocatalytic activity through photogenerated electron shuttling in a Z-scheme manner. Phys Chem Chem Phys.

[42] Siol S, Hellmann JC, Tilley SD, Graetzel M, Morasch J, Deuermeier J, Jaegermann W, Klein A (2016). Band alignment engineering at Cu_2_O/ZnO Heterointerfaces. ACS Appl Mater Interfaces.

[43] Ma LT, Fan HQ, Tian HL, Fang JW, Qian XZ (2016). The n-ZnO/n-In2O3 heterojunction formed by a surface-modification and their potential barrier-control in methanal gas sensing. Sens Actuators B: Chem.

[44] Xu R, Li HH, Zhang WW, Yang ZP, Liu GW, Xu ZW, Shao HC, Qiao GJ (2016). The fabrication of In2O3/In2S3/Ag nanocubes for efficient photoelectrochemical water splitting. Phys Chem Chem Phys.

