# Supplementary material for: Hydrothermal synthesis of In2O3 nanoparticles hybrid twins hexagonal disk ZnO heterostructures for enhanced photocatalytic activities and stability
Source: Nanoscale Res Lett. 2017 Jul 25;12:466. doi: 10.1186/s11671-017-2233-3 (PMC5526824; doi:10.1186/s11671-017-2233-3)
Supplement: Additional file 1: — Figure S1 The temporal evolution of spectral of MO photodecomposition by Zn-In-4. (DOC 571 kb) [file 11671_2017_2233_MOESM1_ESM.doc]

**Supplementary material**

**Hydrothermal synthesis of In_2_O_3_ nanoparticles hybrid twins hexagonal disk ZnO heterostructures for enhanced photocatalytic activities and stability**

Hairui Liu*, Haifa Zhai, Chunjie Hu, Jien Yang, Zhiyong Liu

*College of Physics & Materials science, Henan Normal University, Henan Key Laboratory of Photovoltaic Materials, Xinxiang 453007, PR China.*

| 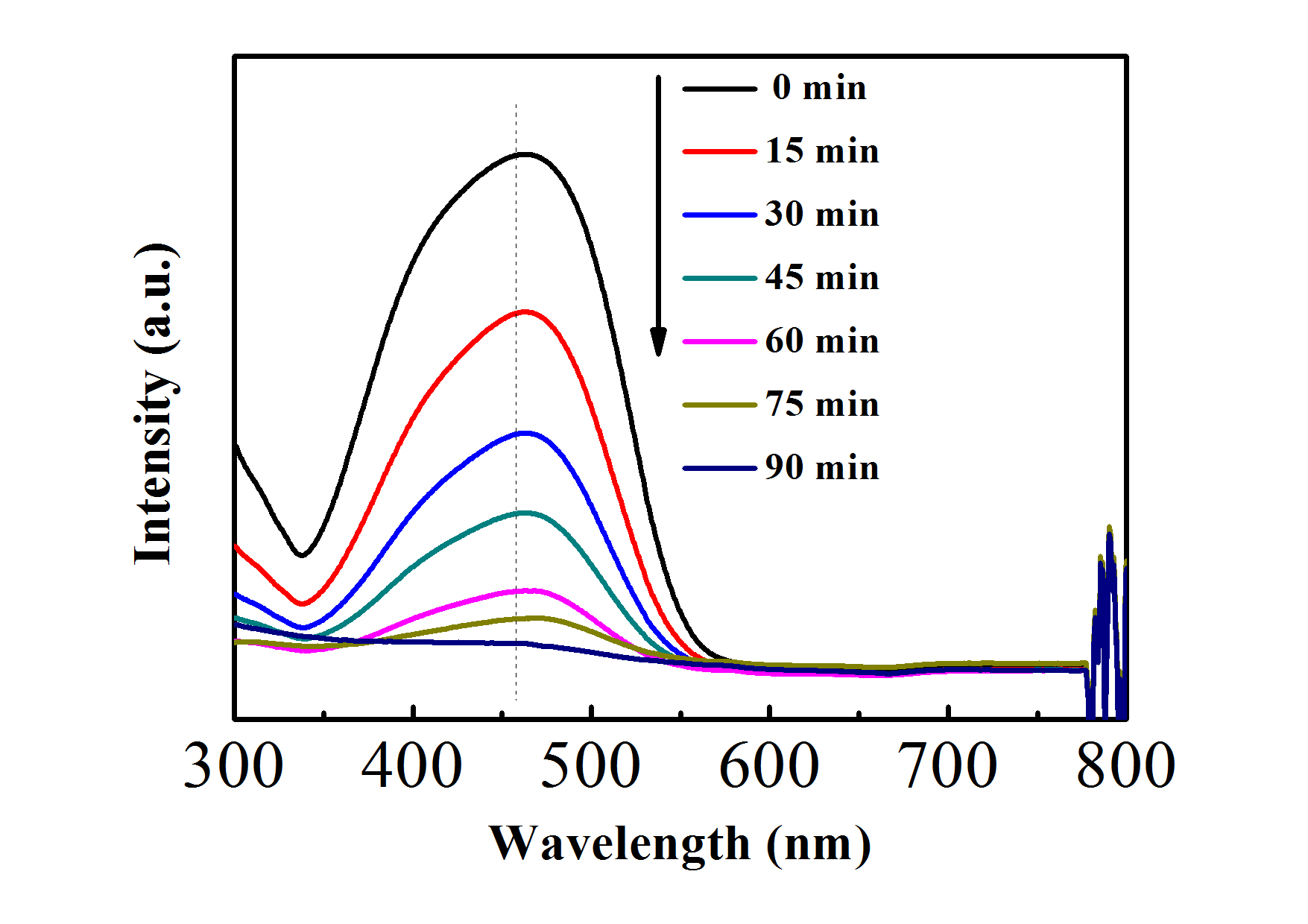 |
| --- |
| Fig. S(1) The temporal evolution of spectral of MO photodecomposition by Zn-In-4. |

The temporal evolution of spectral changes accompanying the photodecomposition of MO over as-prepared Zn-In-4 is shown in Fig. S(1). The characteristic absorption peak intensity of MO at 664 nm gradually decreased with the increase of irradiation time and the color of MO-containing solution was also changed from initial lemon yellow to almost transparent color after 60 min reaction, indicating that the MO have been completely decomposed during the photocatalytic process.
